# Antioxidant Contents in a Mediterranean Population of *Plantago lanceolata* L. Exploited for Quarry Reclamation Interventions

**DOI:** 10.3390/plants11060791

**Published:** 2022-03-16

**Authors:** Federico Sanna, Giovanna Piluzza, Giuseppe Campesi, Maria Giovanna Molinu, Giovanni Antonio Re, Leonardo Sulas

**Affiliations:** 1 National Research Council, Institute for the Animal Production System in Mediterranean Environment, Traversa La Crucca 3, Località Baldinca, 07100 Sassari, Italy; federico.sanna@cnr.it (F.S.); giuseppecampesi2003@gmail.com (G.C.); giovanniantonio.re@cnr.it (G.A.R.); leonardo.sulas@cnr.it (L.S.); 2 National Research Council, Institute of Sciences of Food Production, Traversa La Crucca 3, Località Baldinca, 07100 Sassari, Italy; mariagiovanna.molinu@cnr.it

**Keywords:** plantain, multiple uses, phenolic compounds, verbascoside, seasonal variations

## Abstract

*Plantago lanceolata* L. (plantain) is an interesting multipurpose perennial species whose aerial parts are used in herbal medicine due to its precious phytochemicals and are palatable to animals. Moreover, peculiar traits such as drought tolerance, an extended growth season and a deep root system, make plantain a promising pioneer plant for quarry reclamation based on the use of native species. This study evaluated the effects of different environmental conditions and seasons on the accumulation of the bioactive compounds of its aerial organs. An autochthonous plantain population was grown in three locations in Sardinia (Italy). Leaves, peduncles and inflorescences were collected between October 2020 and July 2021. Phenolic contents and antioxidant capacity were determined. The analysis of the individual phenolic compounds was performed using liquid chromatography. In leaves, the content of total phenolics, antioxidant capacity and total flavonoids were significantly influenced by location and season. Total phenolic content ranged from 65 to 240 g gallic acid equivalent kg^−1^, whereas total flavonoids were from 16 to about 89 g catechin equivalent kg^−1^. Neochlorogenic, chlorogenic, cryptochlorogenic acids, verbascoside, diosmin and luteolin were identified in the methanolic extracts of leaves, peduncles and inflorescences. Verbascoside was the main antioxidant isolated from plantain extracts. Results evidenced an increasing accumulation pattern of phenolics from vegetative stage to flowering, followed by a decrement towards the seed ripening as well as site-specific differences with amounts of phenolics even 25% higher for same plantain accession.

## 1. Introduction

The genus *Plantago* L. (Plantaginaceae) comprises about 275 species, with a cosmopolitan distribution. The aerial organs of Plantago species are used as herbal remedies in the treatment of several diseases related to the respiratory and digestive organs, skin and against various infections [1]. *Plantago lanceolata* L. (narrowleaf plantain, plantain, thereinafter plantain) is common in roadside grassland with a worldwide distribution, as perennial, rarely annual or biennial species, representing an interesting multipurpose plant. It grows in the spontaneous flora of Central Europe and is widely used in folk medicine [2,3] for its valuable source of health-beneficial phytochemicals [4]. Concurrently, plantain is a herb with a broad distribution in grasslands throughout the temperate world. It is popular fodder crop in New Zealand, where the most intensive research on its use and role in permanent grasslands has been carried out [5,6,7]. The leaf is highly palatable to grazing animals, providing a well-appreciated mineral- and protein-enriched forage, which contains a range of biologically active compounds, often in large quantities, to impart anti-oxidative, enzyme inhibitory and anthelmintic activities in farm animals [7,8,9,10,11]. In addition, plantain represents a valuable source of raw material for various industrial applications [12], and preliminary observations indicated its promising utilization as a pioneer plant for revegetation in quarry reclamation interventions [13].

The benefits of plantain aerial parts in cardiovascular diseases and diabetes have been reported and attributed to the presence of luteolin 7-*O*-glucoside, rutin, chlorogenic acid and quercetin hexoside [3].

On the other hand, different types of medicinal herbs have been traditionally used to treat various digestive disorders in production animals [14]. Their effectiveness is based on their antioxidant and antimicrobial properties to modulate the functionality of the ruminant microbiome [15]. In addition, herb secondary metabolites have been commonly used to reduce stress, favor liver function and improve meat quality [16,17]. Reza et al. [8] found that supplementation of the lambs’ diet with plantain showed some beneficial effects on productivity and against parasitic infections. The bioactive components in plantain, notably acteoside (i.e., verbascoside), can ferment rapidly in the rumen resulting in increased gas production and used as an energy source for lamb [18]. Moreover, methane production, one of the major losses of energy from rumen, could be reduced when cows graze on plantain pastures [19].

Anthropogenic activities, such as quarrying or stone crushing, destroy the natural habitat in the vegetation and forest ecosystem, modifying deeply chemical and physical characteristics of the soil. To allow quarry activities, authorities generally require planning details to ensure the subsequent restoration. Adaptive management of quarries could increase biodiversity and vegetation of high conservation value in industrialized and farmed landscapes [20,21,22], and quarry rehabilitation could represent an opportunity to re-establish native grassland species [23,24]. In limestone quarries, restoration ecologists need to develop adequate procedures for establishing native plant communities and the success will depend on improving the substrate condition, selecting efficient species pool for revegetation and preventing invasion by alien species [22,25].

Recovery and re-release of reclaimed areas to the surrounding natural ecosystem can also play an ecological role at the level of wildlife communities (i.e., ungulates) [25].

As a result of injury or shock significant increases in phytochemicals (phenolics, tannins, flavonoids, saponins) were observed in plant samples collected from a quarry site [26]. The same authors referred that the increased phytochemical secretion may be a means of plant adaptation to the induced environmental changes. The oxidative stress may augment the antioxidant production enabling plants to neutralize the free radicals including reactive oxygen species and reactive nitrogen species. Hence, the extent of abiotic stress tolerance might be predicted by the phenolics concentration in plant tissues, which varies greatly in different plant species under an array of external factors and help plants to acclimatize to unfavorable environments [27].

Plant growth, development, biomass accumulation and plant metabolism are usually influenced by phenolic compounds [27]. On the other hand, levels of antioxidants in plantain might vary because of both genetic factors and environmental conditions [28]. Despite there being plenty of literature dealing with medicinal uses, phenolic content and antioxidant activity in *Plantago* spp., very little information is available regarding seasonal variations in phenolic content in different aerial plant organs (e.g., leaves, flowers, fruits, peduncles) as well as in different contexts of utilization of plantain. We hypothesized that different conditions in the growth environment (i.e., cultivation in open field conditions for seed multiplication vs. establishment on an artificial soil substrate for quarry reclamation) might influence the phenolic content in the plant parts of the same plantain accession. In the frame of a general activity aimed at the exploitation of Sardinian herbaceous plant germplasm for traditional and alternative uses, this study evaluated the effects of different environmental conditions and seasons on the accumulation of bioactive compounds of a Mediterranean plantain population, whose seed is specifically produced to be exploited in quarry rehabilitation interventions.

## 2. Results and Discussion

### 2.1. Phenolic Content and Antioxidant Capacity

In leaves, the contents of total phenolics (TotP), non-tannic phenolics (NTP), tannic phenolics (TP), antioxidant capacity and total flavonoids (TotF) were affected significantly by both location and harvest time (Table 1 and Table 2). At Quarry2, leaves showed the highest total phenolic, DPPH antioxidant capacity and total flavonoid values in April and October (Table 1). Overall, the content of TotP in leaves ranged from 65 in Field (July) to 240.3 g gallic acid equivalent (GAE) kg^−1^ in Quarry2 (April). Quarry1 showed the highest values in April and July with the peaks of 230 and 189.3 g GAE kg^−1^, respectively. Quarry2 showed the highest values in April and October, with the values of 240.3 and 190.9 g GAE kg^−1^, respectively (Table 1). Interestingly, at each location results indicated a quite clear seasonal variation in the accumulation of phenolics in the following order April > January > October > July. Such as order well described a trend indicating an increasing accumulation pattern of phenolics from vegetative stages to flowering, when values peaked, followed by a decrement towards the seed ripening and plant senescence phase. For Mediterranean climate areas, featured by high temperatures and drought periods, the evidenced accumulation pattern is in accordance with other authors indicating the highest levels of phenolics at the full-flowering stage even if for herbs other than plantain [29].

A content of total phenolics of 53 mg of tannic acid equivalent 100 g^−1^ DW was found in leaves of plantain grown in Ukraine by Sotek et al. [30]. This value is quite lower than our results, but the extraction was done with water and the samples were not freeze dried. Hamacher et al. [10] found a content of 65 g tannic acid equivalent kg^−1^, but they did not report the plant phenological stages; in addition, the environmental conditions were very different to our study. Kapp-Bitter et al. [31], found a content of 89.9 g kg^−1^, like our result for Field in July, in fact they harvested the plants during the seed ripening stage.

Gligor et al. [32] found in 70% ethyl alcohol extract leaves a content of total phenolics of 45.35 mg GAE mL^−1^ and total flavonoids equal to 26.49 mg QE mL^−1^, similar to our results for TotP at Field in July while the corresponding content of TotF was about 1.6 times higher than our results. However, it must be considered that they performed the extraction with a different solvent. Additionally, they did not indicate the phenological stage at harvest, indeed the content of bioactive compounds is affected by the plant phenological stage [33,34,35,36,37]. A study performed in Bosnia by Ahatović et al. [38] on whole plantain shoot at flowering found a total phenolics content of about 80, 130, 60 mg GAE g^−1^ DW, in a serpentine quarry, an anthropogenic area and from a non-metalliferous soil, respectively. The comparison with our data cannot be done because they analyzed unpartitioned shoots (leaves and inflorescences) and from areas contaminated with heavy metals.

Taken together our findings also evidenced site-specific differences, indicating values up to 25% higher in Quarry2 for the contents of total phenolics, non-tannic phenolics, tannic phenolics and total flavonoids. Additionally, our information is very useful for identifying the best harvest time and/or the best environmental conditions for maximize the yield of phenolics from the same plantain population.

**Figure 1:**
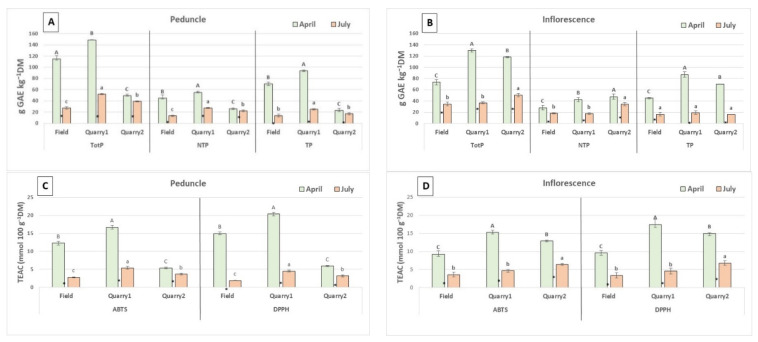
Phenolic content (TotP), non-tannic phenolics (NTP), tannic phenolics (TP) (**A**,**B**) and antioxidant capacity (ABTS, DPPH assays) (**C**,**D**) in peduncle and inflorescence without peduncle constituted by flowers (April) and fruits (July) of plantain. Vertical bars indicate standard deviations of means. Values with the same capital letters for April are not significantly different at *p* ≤ 0.05, values with the same small letters for July are not significantly different at *p* ≤ 0.05. Asterisks between histograms indicate significance for the harvesting month (within the same locations). LSD test for treatment differences. * *p* ≤ 0.05.

There were differences among locations in the content of phenolics and antioxidant capacity in the inflorescence and its peduncle in April and July, the values being lower in July (Figure 1A–D). The higher value of total phenolics in peduncle and inflorescence were recorded in Quarry1 with 148.8 and 129.8 g GAE kg^−1^ DM, respectively. The same trend was observed for antioxidant capacity, the higher values of 20.4 and 17.5 mmol 100 g^−1^ Trolox equivalent antioxidant capacity (TEAC), with DPPH assay, were showed by Quarry1 in peduncle and inflorescence.

**Figure 2:**
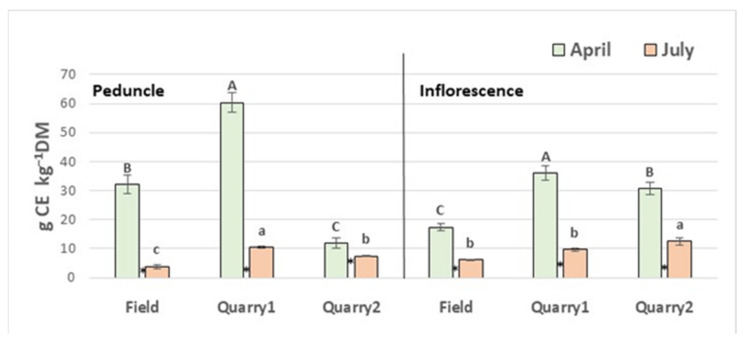
Total flavonoid (TotF) contents in peduncle and inflorescence of plantain in the three locations. Vertical bars indicate standard deviations of means. Values with the same capital letters for April are not significantly different at *p* ≤ 0.05; values with the same small letters for July are not significantly different at *p* ≤ 0.05. Asterisks between histograms indicate significance for the harvesting month (within the same location). LSD test for treatment differences. * p ≤ 0.05.

The content of total flavonoids differed in peduncle and inflorescence in April and July and between locations with the values lower in July (Figure 2).

At each location results indicated a markedly seasonal reduction pattern in the accumulation of phenolics from flowering (April) to seed ripening (July) stage in both peduncle and inflorescence. Moreover, absolute differences in the contents of phenolics in peduncle and inflorescence were also remarkable among locations at the same harvest time.

**Figure 3:**
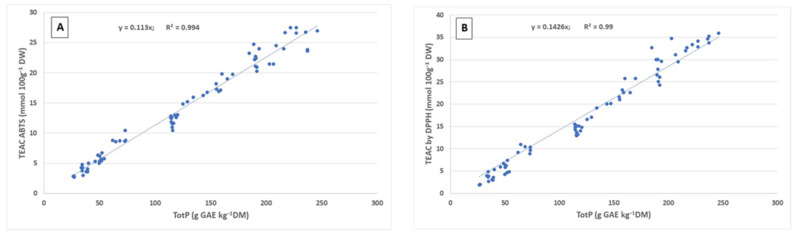
Correlations (R2) established between total phenolics (TotP) and antioxidant capacity ABTS (**A**) and DPPH (**B**) in leaves, peduncles and inflorescences of plantain.

Significant correlations were found between TotP and antioxidant capacity assayed by means ABTS (Figure 3A) and DPPH (Figure 3B) methods. Our data are in agreement with other reports indicating the relationship between total phenolics content and antioxidant capacity [39,40]. Conversely, Sotek et al. [30] did not find in plantain a correlation between TotP and antioxidant capacity with ABTS assay.

No condensed tannins (CT) were detected in the investigated plant organs (leaves, peduncles, inflorescence) and locations. In a study on mature herbs, as a supplement to ruminant diet, CT were not detected in plantain [31], as in our results. Conversely, a CT content of 3 g kg^−1^ was found by Hamacher et al. [10], the content being very low respect to other plant species under study as *Onobrychis viciifolia* (87.6 g kg^−1^).

To our knowledge, this is the first study regarding the content of phenolic compounds of different plantain aerial organs (leaves, inflorescences, peduncles) at different sampling times and contexts of utilization. Additionally, based on the recorded differences regarding the content of phenolic compounds from the same plantain accession in different environment of cultivation, our findings also suggest that quarry revegetation activities could be coupled with the plantain cropping for the subsequent exploitation of its bioactive compound contents. This parallel activity will result in a peculiar and complementary ecosystem service in addition to those services provided by the revegetation process during the quarry rehabilitation.

### 2.2. Reverse Phase-High Performance Liquid Chromatography (HPLC) Analysis of Phenolic Compounds

Among the 35 individual phenolic compounds that were screened, five single compounds were detected in leaf, peduncle and inflorescence methanolic extracts of plantain (Table 3, Table 4, Table 5 and Table 6). These were: neochlorogenic, chlorogenic, criptochlorogenic acids, verbascoside, diosmin and luteolin. Statistical analysis evidenced variations due to locations and harvest time. Our results agree with other study showing that the content of bioactive compounds in plantain leaves change during the growing season (seasonal variation) [41]. The higher content of neochlorogenic and chlorogenic acids in the leaves was detected at Quarry2 in April and at Quarry1 in January with the values of 0.64 mg g^−1^ DM and 1.79 mg g^−1^ DM, respectively (Table 3).

Chlorogenic acid was not detected by Gligor et al. [32] in plantain leaves harvested in Romania. Conversely, they found syringic, cinnamic, caffeic, ferulic acids, rutin, catechin and quercetin that were not detected in our study, but they performed the extraction with 70% ethyl alcohol and the phenological stage was not indicated. In contrast, Bahadori et al. [4] found chlorogenic acid in leaves, buds and flowers of plantain, and Nichita et al. [42] detected chlorogenic acid in plantain leaves with mass spectrometer instrument LCMS. Chlorogenic acid (CGA, 3-CQA) is the most abundant isomer among caffeoylquinic acid isomers (3-, 4-, and 5-CQA), currently known as 5-CQA as per guidelines of IUPAC, demonstrating very valuable actions involving anti-inflammation and antioxidant properties. CGA is an important and biologically active dietary polyphenol, playing several important and therapeutic roles such as antioxidant activity, antibacterial, hepatoprotective, cardioprotective, anti-inflammatory, antipyretic, neuroprotective, anti-obesity, antiviral, anti-microbial, anti-hypertension, free radical scavenger and a central nervous system stimulator. There is also some evidence that CGA may affect the lipid and glucose metabolism in genetically metabolic disorders [43].

At Quarry1, peduncle harvested in April showed the highest content of neochlorogenic acid and verbascoside with the values of 2.42 and 52 mg g^−1^ DM, respectively, whereas the highest content of chlorogenic acid of peduncle was detected at Field in April (Table 5).

Verbascoside was the main individual phenolic compound isolated from plantain leaf, peduncle and inflorescence extracts (Table 4, Table 5 and Table 6). At Quarry2, leaves in April showed the highest value (89.18 mg g^−1^ DM); while at Field leaves showed the lowest value (5.55 mg g^−1^) in July. Generally, the verbascoside content of peduncle and inflorescence decreased from April to July (Table 5 and Table 6).

Janković et al. [1] performed the experiment in the aerial part of the plant without indicating the phenological stage and shoot component and they reported that plantain contained a significantly higher amount of acteoside (i.e., verbascoside) (21.71 mg g^−1^ DW) than other examined species belonging to Plantago genus. This value is quite similar to our results for Quarry1 and Field leaves in October (Table 4).

Verbascoside (also known as acteoside and kusaginin) is a phenylethanoid glycoside: 2-(3,4-dihydroxyphenyl)ethyl-1-*O*-*L*-rhamnopyranosyl-(1-3)-(4-*O*-E-caeoyl)-*D*-glucopyranoside, which is a product of the shikimic acid pathway. There is a growing interest in phenolic compounds for their potential health benefits, such as preventing of cancer and cardiovascular diseases, and even neurodegenerative disorders. Plants with high amounts of verbascoside have traditionally been used for a variety of purposes, including antibacterial, wound-healing, antioxidant, gastroprotective, neuroprotective, cytoprotective, antitumoral and photoprotective effects [44,45]. Worth noting that the administration of verbascoside isolated from plantain, in the experimental model of human intestinal inflammation, improved the histological patterns and clinical symptoms of colitis, downregulated pro-inflammatory IFN-γ (interferon) secretion and inhibited the NADPH-oxidase-connected oxidative burst (indirect antioxidant effect) in the intestinal macrophages [45]. In the plantain inflorescence, verbascoside was the individual phenolic compound more represented, with the higher content in Quarry1 in April (33.05 mg g^−1^ DM) (Table 6). Budzianowska et al. [46] found acteoside in the inflorescence, the chemical structures were established by 1D and 2D NMR and HRESIMS spectral methods.

Janković et al. [1] found a luteolin content of 15.52 µg g^−1^ lower than our results (range 0.04 to 0.47 mg g^−1^). In our study luteolin showed the higher content at Field on leaves and peduncle in July (Table 4 and Table 5).

In the methanolic leaf extract of plantain grown in Iraq, without indicating the phenological stage, caffeic, ferulic, sinapic acids, o-cumaric, rutin, myricetin, quercetin and kaempferol were detected [47]; in contrast, these individual phenolic compounds were not identified in our study. Moreover, they reported antiproliferative effects against CAL51 triple-negative breast cancer cells and showed minor effect on the other breast cancer cell types studied.

Diosmin was only detected in the inflorescence of April with the values of 13.2 ± 0.2, 19.3 ± 0.42, 39.4 ± 2.39 at Field, Quarry1 and Quarry2, respectively, with statistically significant differences.

Diosmin, 3′,5,7-trihydroxy-4′-methoxyflavone-7-rutinoside, is a flavone naturally occurring as flavonoid glycoside that can be isolated from various plant sources belonging to Citrus spp., legumes and olive leaves. Upon ingestion, diosmin is extensively converted to the aglycone diosmetin, an O-methylated flavone (3′,5,7-trihydroxy-4′-methoxyflavone), by intestinal bacteria and absorbed as such in the organism. Studies have shown that diosmin may have a preventive effect on several diseases such as hyperglycemia, hyperlipidemia, antiulcer and antiplatelet activity, but the most relevant therapeutic use of diosmin is in the treatment of chronic venous insufficiency and hemorrhoids [48].

A study performed in plantain leaves with liquid chromatography-tandem mass spectrometry assay did not find rutin, quercetin, diosmin and kaempferol as our results [49].

Interesting, Li et al. [29] evidenced the seasonal variation of the bioactive compounds that depending on region, habitat, altitude, species-specific, organ-specific and seasonal variation might differ due to plant age. In accordance with Li et al. [29], our research evidenced important variations in the contents of bioactive compounds associated with seasonal variations, plant tissues and environments.

Pol et al. [7] highlighted that the use of plantain leaves as a substitute for hay in sheep nutrition represented a valuable addition to livestock feed, characterized by above-average health. As supported by our findings, the advantages of plantain as a crop lies in its high content of valuable bioactive compounds, which are beneficial for human and animal consumption. On the other hand, in our study, the recovery is carried out in quarries whose surrounding areas are normally grazed by both wildlife and reared ruminants. Undoubtedly, the establishment of plantain might significantly improve the pasture quality of these surrounding areas.

## 3. Materials and Methods

### 3.1. Plant Material

**Figure 4:**
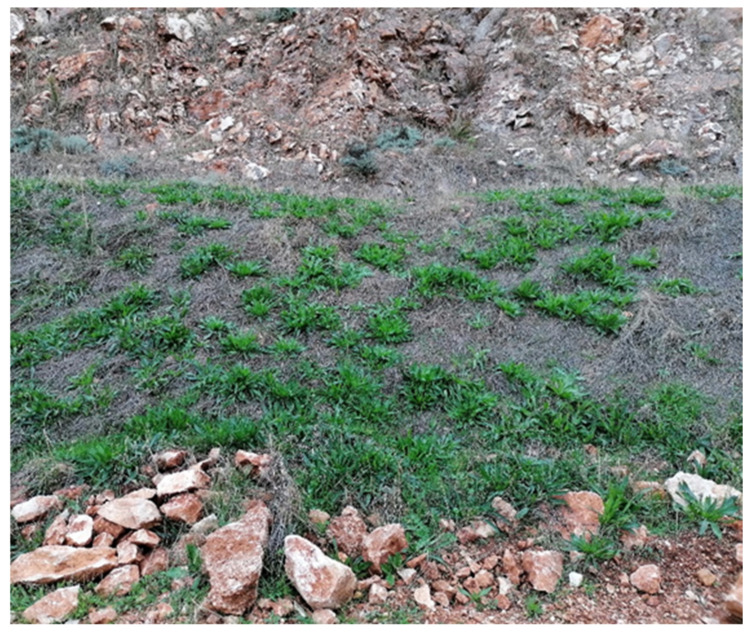
Plantain at Quarry2 site.

Previously, a germplasm collection was carried out in Sardinia and 5 local populations of plantain were investigated for biometric traits and seed production [50]. The population PL001 (Voucher specimens are deposited at the ISPAAM SS) was grown for seed multiplication at the experimental field of CNR-ISPAAM allowing its concurrent use in three locations of Sardinia (Italy) (Table 7), namely in open field (Field) for seed production and in quarries (Quarry1 and Quarry2) for reclamation interventions (Figure 4).

The aerial parts of nine plantain plants having the same stand age (three-year old) were harvested in October 2020, January, April and July 2021, respectively. In the same order, the aforementioned harvest times corresponded to the following stage of development: early vegetative, vegetative, flowering, ripened seeds and senescence. The aerial parts were represented by leaves, peduncles (separated from inflorescences) and inflorescences without peduncles, the latter constituted by flowers or fruit, which were harvested in April and July, respectively. The samples were immediately frozen in liquid nitrogen and then freeze dried with Heto Lyolab 3000 (Heto-Holten A/S, Allerod, Denmark).

### 3.2. Sample Preparation

Phenolic compounds were evaluated in freeze dried samples, ground to a fine powder for the chemical analysis as reported by Piluzza et al. [51]. At harvest, leaf and inflorescence samples were frozen and stored at −20 °C, until lyophilization with Heto Lyolab 3000 (Heto-Holten A/S, Allerød, Denmark) for 48 h (−55 °C). The powdered material was then used for extract preparations as reported by Molinu et al. [52]. Ground leaf subsamples (50 mg) were extracted with 2.5 mL methanol/water (8:2 *v*/*v*) mixture and shaken for 24 h. The extracts were then centrifuged for 10 min at 3900 rpm and the supernatant was stored at −20 °C until analysis. Samples were analyzed in triplicate.

### 3.3. Phenolic Content and Antioxidant Capacity

Total phenolics (TotP), non-tannic phenolics (NTP) and tannic phenolics (TP) of extracts were determined using the Folin–Ciocalteau reagent, according to procedures previously described [53]. Results were reported as g of gallic acid equivalent (GAE) kg^−1^ dry matter of plant material (g GAE kg^−1^ DM) by means of a calibration curve of gallic acid. Total flavonoids (TotF) were quantified by the AlCl_3_ method, following procedures previously reported [54]. TotF were quantified by catechin calibration curve and results were reported as g of catechin equivalent (CE) kg^−1^ dry matter (g CE kg^−1^ DM).

The butanol assay was used for quantification of the extractable condensed tannins content, reported as g delphinidin equivalent per kg^−1^ dry matter (g DE kg^−1^ DM) [54].

Antioxidant capacity was evaluated by ABTS ((2,2′-azinobis (3-ethylbenzothiazoline-6-sulphonic acid) diammonium salt)) and by DPPH (1,1-diphenyl-2-picrylhydrazyl) assays [51]. Trolox (6-hydroxy-2,5,7,8-tetramethylchroman-2-carboxylic acid), was used as the reference standard. The results were expressed in terms of Trolox equivalent antioxidant capacity (TEAC), as mmol Trolox equivalents 100 g^−1^ dry weight of leaves (mmol TEAC 100 g^−1^ DW).

### 3.4. RP-HPLC Analysis of Phenolic Compounds

Analyses of individual phenolic compounds were carried out using an Agilent 1260 series HPLC instrument (Agilent Technologies, Palo Alto, CA, USA) equipped with a quaternary pump, degasser, column thermostat, auto-sampler and diode array detector. Chromatographic separation was carried out according to Molinu et al. [52]. The column was a Zorbax Eclipse plus C18 (250 × 4.6 mm, 5 µm; Agilent). Column temperature was set to 30 °C and the flow rate was 0.8 mL min^−1^. The injection volume was 10 μL and the detection wavelengths were set to 280, 330 and 350 nm. Data were processed using the Agilent OpenLAB CDS ChemStation edition 2012. The concentration of six phenolic compounds (neoclorogenic acid, clorogenic acid, criptoclorogenic acid, verbascoside, diosmin, luteolin) were carried out comparing their UV-vis spectra and retention times with those of analytically pure standard.

### 3.5. Data Analyses

Laboratory measurement values were subjected to a two-way analysis of variance, using Statgraphics Centurion XVI version (StatPoint Technologies Inc., Warrenton, VA, USA, 2009), to test the effects of location, harvest time and their interaction on the following variables: concentrations for antioxidant capacity, total phenolics, total flavonoids and individual phenolic compounds. Differences between means were assessed with the Fisher’s least significant difference (LSD) procedure for means separation. The significance level was fixed at *p* ≤ 0.05 for all the statistical analyses.

## 4. Conclusions

Our study supported new insights into seasonal variations of phenolic content, antioxidant activity and quantification of individual phenolic compounds from aerial plant organs of a Mediterranean plantain accession grown in different environmental conditions, from open field for seed multiplication to quarries for reclamation interventions. Overall results evidenced important variations in phenolic compound contents and antioxidant capacity associated with aerial plant organs, seasonal variations and cultivation environments. Our research showed that the content of phenolic compounds and the antioxidant capacity in leaves, peduncles and inflorescences were affected significantly by season and location. An evident trend indicating an increasing accumulation pattern of phenolics from vegetative stage to flowering, followed by a decrement towards the seed ripening and plant senescence was achieved. Additionally, a significant effect of the growth environmental conditions on the accumulation of plantain bioactive compounds was demonstrated. In fact, Quarry2 showed values up to 25% higher than remaining locations for the leaf contents of total phenolics, non-tannic phenolics, tannic phenolics and total flavonoids. Our findings also identified the best harvest time and environmental conditions for maximizing the yield of phenolics from the same plantain accession, suggesting the possibility of combining quarry revegetation activities with the concurrent production and exploitation of bioactive compounds from plantain.

Verbascoside was the main antioxidant isolated from plantain extracts confirming previous studies. As verbascoside, chlorogenic acid and other phenolic compounds identified in this study have been found from the literature to have several pharmacological activities, the results might be useful in subsequent studies to evaluate their biological activity.

## Figures and Tables

**Table 1:** Total phenolics (TotP), non-tannic phenolics (NTP), tannic phenolics (TP) in leaves of the examined plantain.

	TotP (g GAE kg^−1^ DW)				NTP (g GAE kg^−1^ DW)				TP (g GAE kg^−1^ DW)			
	October	January	April	July	October	January	April	July	October	January	April	July
Field	116.2 Bd	157.3 Cb	190.9 Ca	65.2 Cc	49.5 Bb	45.8 Bc	84.2 Ba	26.2 Bd	66.7 Bc	111.6 Ca	106.7 Cb	39.0 Cd
Quarry1	116.6 Bd	218.3 Ab	230.0 Ba	189.3 Ac	52.8 Bd	61.9 Ac	108.5 Aa	78.3 Ab	63.8 Bd	156.4 Aa	121.6 Bb	110.9 Ac
Quarry2	190.9 Ad	206.3 Bb	240.3 Aa	165.1 Bc	65.7 Ac	58.9 Ad	104.9 Aa	79.4 Ab	125.2 Ac	147.4 Ba	133.7 Ab	85.7 Bd
G × H	**				**				**			

At each column of harvest time, means followed by the same capital letters are not significantly different at *p* ≤ 0.05; In the row, means followed by the same small letters are not significantly different at *p* ≤ 0.05 within locations. G × H: interaction between locations and harvest time. ns, not significant; ** *p* ≤0.001.

**Table 2:** Trolox Equivalent Antioxidant Capacity (TEAC) by ABTS and DPPH methods and total flavonoids (TotF) in leaves of the examined plantain.

	TEAC (mmol 100 g^−1^ DW)								TotF (g CE kg^−1^ DW)			
	ABTS				DPPH							
	October	January	April	July	October	January	April	July	October	January	April	July
Field	10.9 Bc	17.4 Cb	21.5 Ca	8.7 Cd	13.0 Bd	22.4 Bb	27.3 Ba	10.2 Cc	35.1 Bc	55.4 Cb	66.9 Ca	16.3 Cd
Quarry1	12.2 Bc	26.0 Aa	26.9 Aa	23.9 Ab	13.9 Bc	32.6 Aab	33.8 Aa	30.7 Ab	37.2 Bc	78.9 Aa	81.2 Ba	60.4 Ab
Quarry2	21.6 Ab	22.4 Bb	24.8 Ba	19.5 Bc	25.8 Ac	31.7 Ab	34.9 Aa	24.7 Bc	67.0 Ac	74.0 Bb	89.6 Aa	43.1 Bd
G × H	**				**				**			

At each column of harvest time, means followed by the same capital letters are not significantly different at *p* ≤ 0.05; In the row, means followed by the same small letters are not significantly different at *p* ≤ 0.05 within locations. G × H: interaction between locations and harvest time. ns, not significant; ** *p* ≤ 0.001.

**Table 3:** HPLC analysis of phenolic acids (mg g^−1^ DM) in leaves of the examined plantain. (Means ± SD, *n* = 3, LSD test).

	Neochlorogenic Acid				Chlorogenic Acid				Cryptochlorogenic Acid			
t_R_, min	9.46				11.27				11.57			
	October	January	April	July	October	January	April	July	October	January	April	July
Field	0.18 Bc	0.27 Bb	0.52 Ba	0.06 Cd	0.70 Bc	1.38 Ca	1.31 Ba	1.04 Bb	0.16 Ab	0.12 Ac	0.25 Aa	0.13 Bc
Quarry1	0.17 Bd	0.34 Ab	0.41 Ca	0.29 Ac	0.70 Bc	1.79 Aa	1.27 Bb	1.32 Ab	0.14 Ac	0.13 Ac	0.26 Aa	0.20 Ab
Quarry2	0.25 Ab	0.26 Bb	0.64 Aa	0.25 Bb	0.98 Ab	1.51 Ba	1.49 Aa	1.02 Bb	0.13 Bbc	0.14 Ab	0.20 Ba	0.12 Bc
G × H	**				**				**			

At each column of harvest time, means followed by the same capital letters are not significantly different at *p* ≤ 0.05; In the row, means followed by the same small letters are not significantly different at *p* ≤ 0.05 within locations. G × H: interaction between locations and harvest time. ns, not significant; ** *p* ≤ 0.001. t_R_: retention time.

**Table 4:** HPLC analysis of phenolic compounds (mg g^−1^ DM) in leaves of the examined plantain. (Means ± SD, *n* = 3, LSD test).

	Verbascoside				Luteolin			
t_R_, min	21.19				33.73			
	October	January	April	July	October	January	April	July
Field	24.40 Bc	45.13 Cb	62.27 Ca	5.55 Cd	0.06 b	tr	nd	0.47 Aa
Quarry1	20.49 Cd	82.68 Aa	79.88 Bb	41.94 Ac	nd	0.09 a	nd	0.06 Bb
Quarry2	50.71 Ac	62.47 Db	89.18 Aa	30.21 Bd	nd	tr	nd	0.04 C
G × H	**				**			

At each column of harvest time, means followed by the same capital letters are not significantly different at *p* ≤ 0.05; In the row, means followed by the same small letters are not significantly different at *p* ≤ 0.05 within locations. G × H: interaction between locations and harvest time. ns, not significant; ** *p* ≤ 0.001; tr: trace quantities, nd: not detected. t_R:_ retention time.

**Table 5:** HPLC analysis of phenolic compounds (mg g^−1^ DM) in peduncle of the examined plantain. (Means ± SD, *n* = 3, LSD test).

	Neochlorogenic Acid		Chlorogenic Acid		Cryptochlorogenic Acid		Verbascoside		Luteolin	
t_R_, min	9.46		11.27		11.57		21.19		33.73	
	April	July	April	July	April	July	April	July	April	July
Field	1.63 B	tr	4.32 Aa	0.58 Cb	nd	0.06 B	35.37 Ba	1.20 Cb	tr	0.49 A
Quarry1	2.42 Aa	0.27 Ab	3.03 Ba	1.22 Ab	nd	0.22 A	52.00 Aa	2.60 Ab	nd	0.28 B
Quarry2	0.36 Ca	0.13 Bb	1.52 Ca	0.87 Bb	nd	nd	3.80 Ca	1.64 Bb	0.08 a	0.04 Cb
G × H	**		**				**		**	

At each column of harvest time, means followed by the same capital letters are not significantly different at *p* ≤ 0.05; In the row, means followed by the same small letters are not significantly different at *p* ≤ 0.05 within locations. G × H: interaction between locations and harvest time. ns, not significant; ** *p* ≤ 0.001; tr: trace quantities, nd: not detected. t_R_: retention time.

**Table 6:** HPLC analysis of phenolic compounds (mg g^−1^ DM) in inflorescence without peduncle, constituted by flowers in April and fruits in July, of the examined plantain. (Means ± SD, *n* = 3, LSD test).

	Neochlorogenic Acid		Chlorogenic Acid		Cryptochlorogenic Acid		Verbascoside		Luteolin	
t_R_, min	9.46		11.27		11.57		21.19		33.73	
	April	July	April	July	April	July	April	July	April	July
Field	0.09 B	tr	1.12 B	nd	nd	nd	9.80 Ca	1.29 Bb	tr	nd
Quarry1	0.21 A	tr	1.17 A	tr	nd	tr	33.05 Aa	2.14 ABb	tr	nd
Quarry2	0.08 C	tr	1.07 C	nd	nd	nd	11.80 Ba	2.89 Ab	tr	nd
G × H	**		**				**			

At each column of harvest time, means followed by the same capital letters are not significantly different at *p* ≤ 0.05; In the row, means followed by the same small letters are not significantly different at *p* ≤ 0.05 within locations. G × H: interaction between locations and harvest time. ns, not significant; ** *p* ≤0.001; tr: trace quantities, nd: not detected. t_R_: retention time.

**Table 7:** Main soil characteristics and land use of the three experimental locations in Sardinia (Italy).

Locations	Leccari, Sassari	Abba Mejga, Sassari	Sas Funtanas, Nuoro
Short name	Field	Quarry1	Quarry2
Latitude, Longitude	40°45′18″ N, 8°24′59″ E	40°45′12″ N, 8°24′25″ E	40°33′58″ N, 9°39′49″ E
Altitude (m a.s.l.)	27	40	600
Soil series (FAO, 2006)	Eutric, Calcaric and Mollic Fluvisols	Eutric and Lithic Leptosol	Lithic Xerorthents
Land use	CNR experimental field	Limestone quarry	Limestone quarry
Current activity	Seed production	Reclamation	Reclamation
Sand/Silt/Clay (%)	68/12/20	40/30/30	62/23/27
pH	8.1	8.3	7.8
Organic C (g kg^−1^)	0.8	0.9	0.5
Total N (g kg^−1^)	0.9	0.1	0.9
Assimilable P (mg kg^−1^)	20.3	0	0.7

## Data Availability

The data presented in this study are available on request from the authors.
